# Prevalence and socio-demographic correlates of physical activity levels among South African adults in Cape Town and Mount Frere communities in 2008-2009

**DOI:** 10.1186/s13690-016-0167-3

**Published:** 2016-12-29

**Authors:** Pasmore Malambo, Andre P. Kengne, Estelle V. Lambert, Anniza De Villiers, Thandi Puoane

**Affiliations:** 1 University of Western Cape, School of Public Health, Robert Sobukwe Road, Bellville, Cape Town 7535 South Africa; 2 Non-communicable disease Unit, South African Medical Research Council, Francie van Zijl Drive, Parowvallei, P.O. Box 19070, 7505 Tygerberg, Cape Town South Africa; 3 Division of Exercise Science and Sports Medicine, Department of Human Biology, Faculty of Health Sciences, University of Cape Town, P.O. Box 115, Newlands, 7725 Cape Town South Africa

**Keywords:** Physical activity, Socio-demography, Determinants, Rural, Urban, Adult, Non-communicable diseases, South Africa

## Abstract

**Background:**

Physical activity has been linked to reduced risk of various cardiometabolic disease, cancer, and premature mortality. We investigated the prevalence and socio-demographic correlates of physical activity among adults in urban and rural communities in South Africa. Methods: This was a cross-sectional survey comprising 1733 adults aged ≥35 years from the Cape Town (urban) and Mount Frere (rural) sites of the Prospective Urban Rural Epidemiology study. Physical activity was assessed using the validated International Physical Activity Questionnaire. Multinomial logistic regressions were used to relate physical activity with socio-demographic characteristics.

**Results:**

Overall, 74% of participants engaged in moderate-to-vigorous physical activity. In the adjusted regression models, women were 34% less likely to engage in vigorous physical activity (OR =0.66, 95%-CI = 0.47-0.93). Physical activity decreased with age, varied with marital status, education and occupation, always in differential ways between urban and rural participants (all interactions *p* ≤ 0.047). For instance, in urban settings, those with secondary education were more likely to engage in moderate physical activity (OR = 2.06, 95%-CI = 1.08-3.92) than those with tertiary education. Single people were more likely to engage in high physical activity (OR = 2.10, 95%-CI = 1.03-4.28) than divorced. Overall, skilled participants were more likely to engage in vigorous physical activity (OR = 2.07, 95%-CI = 1.41-3.05) driven by significant effect in rural area (OR = 2.70, 95%-CI = 1.51-4.83). Urban participants were more likely to engage in moderate physical activity (OR = 1.67, 95%-CI = 1.31-2.13) than rural participants.

**Conclusions:**

To prevent chronic diseases among South Africans, attention should be paid to specific policies and interventions aimed at promoting PA among young adults in rural and urban setting, and across the social-economic diversity.

## Background

The health benefits of physical activity (PA) in the prevention and control of non-communicable diseases (NCDs) are well established [1]. Participation rates in PA, however, remain low in all age groups [2]. For example, more than 60% of adults worldwide do not reach recommended 150 min weekly of moderate PA required to be of benefit to their health [3, 4]. It is estimated that physical inactivity defined as any activity insufficient to meet current global recommendations [5], indirectly causes 9% of premature mortality; it was linked to approximately 1.3 million of the 57 million deaths that occurred worldwide in 2008 [4]. For instance, physical inactivity could account for 6% of coronary heart disease, 7% of type 2 diabetes and 10% of cancer [4], making it the fourth leading cause of NCDs [3]. In South Africa, 3.3% of all deaths in 2000 were attributable to PA, ranking it 9^th^ among other risk factors [6]. The prevalence of self-reported physical inactivity is high in both developed countries like the United States, where 32% of adults are physically inactive [7], and in developing countries such as South Africa were 45% adults were reported to be inactive [8]. Other African countries also report a high prevalence of physical inactivity among adults, 49.1% and 52.6% in Swaziland and Mauritania respectively [8].

Similar to other developing countries, South Africa is currently undergoing nutritional, lifestyle, and socioeconomic transitions, with increases in the occurrence of NCDs [9]. Non-communicable diseases of lifestyle share similar modifiable risk factors, which include hypertension, tobacco smoking, diabetes, obesity, hyperlipidaemia and physical inactivity [10]. Physically inactivity in global populations represents a major public health challenge [4]. Documented research comparing activity levels in urban and rural settings suggests that rural adults tend to be less active than their urban counterparts, although findings have been inconsistent [11]. In the USA, a study revealed that PA levels were higher in urban areas than in rural areas [12]. Similarly, in South Africa, subjects in isolated rural areas were found to be more inactive than their urban-dwelling counterparts [10]. Again, similar results were also reported in a study of Kenyan adolescents [13]. Conversely, a South African Demographic and Health Survey found that urban youths were more likely to be physically inactive than rural ones [14].

The importance of promoting physical activity in populations is reflected by the South Africa National Strategic Plan for the Prevention and Control of non-communicable diseases, which targets a 10% reduction in the prevalence of inactivity by 2020 [15]. Indeed, a recommendation for every adult to accumulate 30 min or more of moderate-intensity physical activity on as many days and, preferably, every day of the week [16] is estimated to increase the life expectancy of the world’s population [4].

However, in order to understand PA patterns and make positive changes to them, it is important to understand the independent contributions of urban-rural and socio-demographic risk factors [17]. Conducting population-based studies on prevalence of physical activity and its determinants is necessary to identify the relevant areas in local environments that need change, areas where currently such information is scarce [18]. In this study, we determine the prevalence of self-reported PA and associated socio-demographic factors among South African adults in urban and rural communities.

## Methods

### Study population

This cross-sectional study uses data from the Cape Town (urban) and Mount-Frere (rural) sites of the global Prospective Urban and Rural Epidemiology study (PURE) study. PURE is a multinational cohort study that tracks societal influences, risk factors and chronic non-communicable diseases in urban and rural areas across 17 countries including South Africa. PURE collects baseline data on countries’ characteristics (e.g. economic environments), communities (e.g. nutritional environment), households (e.g. income) and individual determinants (e.g. diet and physical activity) [19]. During a baseline evaluation conducted by PURE in 2008-2009, a representative random sample of adults was selected from well-established rural (Mount Frere) and urban (formal settlements in Cape Town) communities in South Africa. The household inclusion criteria were: (1) to have at least one member who was aged 35-70 years, (2) to be within an identified neighbourhood and (3) to not have members with a disability that precluded walking.

### Data collection

Participants were interviewed in the language of their choice. We used structured, socio-demographic and lifestyle questionnaires that were developed and standardized for the international PURE study [19]. Physical examination included anthropometric measures (height, weight, waist and hip circumference) [20]. In all consenting and eligible individuals, the long version of the International Physical Activity Questionnaire (IPAQ) was used to measure self-reported PA [21].

### Socio-demographic characteristics

We recorded socio-demographic information, specifically: age, sex, marital status, education level, and occupation from each participant. We grouped them into four age categories: 35 to 44 years, 45 to 54 years, 55 to 64 years and 65 years or older. Marital status was classified as never married, currently married, and widowed/divorced/separated. Education level was classified as primary, secondary and tertiary education. Occupational status ranged from 1 to 11 items prompted by the following statement; “*Please indicate which group best describes your main occupation*”. In this study, the occupation status was then categorized as skilled (technicians, machine operators, clerks, skilled agriculture and fishery workers) and less skilled (homemaker, service, shop and market workers).

### Physical activity measure

The IPAQ includes questions on frequency and duration of vigorous and moderate intensity physical activities, and walking in terms of the frequency (days/week) and duration (min/day) in the last 7 days. The physical activities were classified into the domains of work-related, transport-related, household-related and leisure activity for each category of walking, moderate and vigorous-intensity. Weekly minutes of walking, moderate-intensity and vigorous-intensity activity were calculated separately by multiplying the number of days/week by the duration on an average day. In this study, physical activity levels were classified as low, moderate, or high intensity, defined by the IPAQ core group (http://www.ipaq.ki.se) as follows: Low - no activity or some activity reported, but not enough to satisfy the requirements of the other activity categories; Moderate - any of the following 3 criteria: (a) 3 or more days of vigorous-intensity activity for at least 20 min per day, (b) 5 or more days of moderate intensity activity or waking for at least 30 min per day, or (c) 5 or more days of any combination of walking, moderate intensity, or vigorous-intensity activities achieving a minimum of 600 MET-minutes per week; Vigorous - either of the following 2 criteria: (a) 3 or more days of vigorous-intensity activity accumulating at least 1500 MET-minutes per week or (b) 7 days of any combination of walking or moderate- or vigorous intensity activities achieving a minimum of 3000 MET-minutes per week. Acceptable reliability and validity of IPAQ has been reported elsewhere [22].

### Statistical analysis

The starting sample comprised 2064 participants of whom 316 were excluded for unacceptable levels of missing data [23]. A further 15 participants aged less than 35 years were excluded, making a final analytic sample of 1733 participants. We used SPSS® version 22 for Windows (IBM Corp: Armonk New York) for all statistical analyses. Chi squared tests were used to compare socio-demographic characteristics and physical activity. We used multinomial logistic regressions to investigate the determinants of physical activity, with low physical activity as the reference, both in univariate and multivariate models. The differential effects of socio-demographic characteristics on physical activity levels according to urban and rural setting were assessed through interaction tests. Statistical significance was set at *p* < 0.05.

## Results

### General characteristics and pattern of physical activity

Table 1 shows the overall and site-specific (urban/rural), socio-demographic characteristics and the prevalence of PA among the participants. Participants were evenly divided between urban (50.6%) and rural (49.4%) sites. Women comprised the majority of the sample in rural (76.8%) and urban (71.4%) sites and overall (74%) and their proportional was significantly higher than that of men (*p* = 0.011). The most common age group was 45-54 years (33.1%), and age distribution did not differ across sites (*p* = 0.375). Obesity (BMI ≥ 30 kg/m^2^) was higher in the urban site at 57%, versus 42% in the rural site and differences were significant (*p* < 0.001). There were significant differences between urban and rural sites for categories of education, marital status and occupation (all *p* < 0.001), Table 1. Patterns of PA were 31%, 51.5% and 17.5% in the rural site; 20.9%, 61.2% and 17.9% in the urban site and 25.9%, 56.4% and 17.7% in combined sites for low, moderate and vigorous PA respectively in each case, and differences between sites were significant (*p* < 0.001), Table 1.

**Table 1:** Socio-demographic characteristics of adults South Africans from Cape Town and Mount Frere communities in 2008-2009

Variables	Urban (*n* = 877)	Rural (*n* = 856)	*P*-value	Overall (*N* = 1733)
	(%)	(%)		(%)
Gender			0.011	
Female	71.4	76.8		74.0
Male	28.6	23.2		26.0
Age			0.375	
35-44	30.1	31.5		30.8
45-54	35.0	31.1		33.1
55-64	26.0	27.6		26.8
65 and above	8.9	9.8		9.3
BMI (kg/m^2^)			<0.001	
< 18.5	3.7	2.8		3.2
18.5-24.9	21.1	28.2		25.3
25.0-29.9	18.2	27.3		23.6
> 30.0	57.0	41.7		47.9
Education level			<0.001	
Primary	23.8	49.9		36.7
Secondary	69.1	47.3		58.3
Tertiary	7.1	2.8		5.0
Marital status			<0.001	
Single	51.2	30.8		41.1
Currently married	33.6	44.7		39.1
Widowed/divorced/separated	15.2	24.4		19.7
Occupation			<0.001	
Skilled	22.2	14.6		18.5
Less skilled	77.8	85.4		81.5
Ethnicity			0.693	
African	98.7	98.9		98.8
Coloured	1.3	1.1		1.2
Physical activity levels			<0.001	
Low	20.9	31.0		25.9
Moderate	61.2	51.5		56.4
Vigorous	17.9	17.5		17.7

### Socio-demographic characteristics and physical activity levels

Table 2 shows the PA patterns for each socio-demographic category. Overall, gender, age, education level, marital status, occupation and location were significantly associated with physical activity (*p* < 0.01). In stratified analysis, the pattern of PA differed between men and women in the rural site (*p* = 0.031), but not the urban site (*p* = 0.371). The prevalence of vigorous physical activity decreased with increased age group in rural area (*p* < 0.001), and a borderline difference in the distribution of PA across age groups in the urban site (*p* = 0.057). Education levels in urban area was positively associated with vigorous PA (*p* = 0.009), but not in the rural one (*p* = 0.161). This pattern was different for marital status in the urban site (*p* = 0.010), but not in the rural site (*p* = 0.086). The pattern with occupation was mostly similar and differences were significant when stratified by site (*p* < 0.001 in both sites), Table 2.

**Table 2:** Socio-demographic characteristics by physical activity levels among adults South Africans from Cape Town and Mount Frere communities in 2008-2009

Variables	Urban (*n* = 877)				Rural (*n* = 856)				Overall (*N* = 1733)			
	Physical activity				Physical activity				Physical activity			
	Low	Moderate	Vigorous	*P*-value	Low	Moderate	Vigorous	*P*-value	Low	Moderate	Vigorous	*P*-value
	(%)	(%)	(%)		(%)	(%)	(%)		(%)	(%)	(%)	
Gender				0.371				0.031				0.014
Women	21.4	61.8	16.8		32.1	52.2	15.7		26.9	56.9	16.2	
Men	19.5	59.8	20.7		27.1	49.2	23.6		22.9	55.1	22.0	
Age				0.057				<0.001				<0.001
35-44	20.5	62.1	17.4		25.6	48.8	25.6		23.0	55.4	21.5	
45-54	19.9	59.0	21.2		27.1	57.1	15.8		23.2	58.1	18.7	
55-64	18.9	63.6	17.5		33.1	53.0	14.0		26.1	58.2	15.7	
65 and above	32.1	60.3	7.7		54.8	38.1	7.1		43.8	48.8	7.4	
BMI (kg/m^2^)				0.850				0.314				0.463
< 18.5	29.4	52.4	17.6		21.1	63.0	15.9		25.0	58.3	16.7	
18.5-24.9	20.8	57.3	21.9		29.5	52.6	17.9		26.6	54.2	19.2	
25.0-29.9	20.5	61.4	18.1		33.7	55.4	10.9		29.6	57.3	13.1	
> 30.0	17.3	63.5	19.2		32.4	49.1	18.5		25.1	56.0	18.9	
Education level				0.009				0.167				0.003
Primary	24.4	63.6	12.0		33.5	50.8	15.7		30.5	55.0	14.5	
Secondary	18.6	61.9	19.5		29.4	51.6	19.0		22.9	53.8	19.3	
Tertiary	30.6	46.8	22.6		12.5	62.5	25.0		25.6	51.2	23.3	
Marital status				0.010				0.086				<0.001
Single	17.1	61.7	21.2		28.8	51.1	20.1		21.5	57.8	20.8	
Married	23.1	61.7	15.3		29.2	52.0	18.8		26.5	56.2	17.3	
Divorced/separated	28.6	58.6	12.8		36.8	51.2	12.0		33.6	54.1	12.3	
Occupation				<0.001				<0.001				<0.001
Skilled	21.0	51.8	27.2		20.0	48.0	32.0		20.6	50.3	29.1	
Less skilled	20.8	63.9	15.2		32.8	52.1	15.0		27.0	57.8	15.1	
Location												<0.001
Urban									20.9	61.2	17.9	
Rural									31.0	51.5	17.5	

### Multivariable regression analysis and interaction tests

Table 3 shows odds ratios from age and sex-adjusted multinomial regression analyses of socio-demographic characteristics and PA. In these models, when applied to all participants, age (*p* < 0.001), occupation (*p* < 0.001),) and location (*p* < 0.001) were significantly associated with PA level, while there was a borderline association with gender (*p* = 0.055), and no association with education (*p* = 0.116) or marital status (*p* = 0.126), Table 3. With the exception of gender (*p* = 0.072), significant interactions were observed between location and socio-demographic characteristics (results not shown in the table), in their relationship with PA (*p* < 0.001 for age*location, *p* = 0.012 for education level*location, *p* < 0.001 for marital status*location, *p* < 0.001 for occupation*location interaction tests).

**Table 3:** Multinomial logistic regression result of socio-demographic characteristics by physical activity levels with reference to low physical activity in adults South Africans from Cape Town and Mount Frere communities in 2008-2009

Variables	Urban (*n* = 877)				*p*-value	Rural (*n* = 856)				*p*-value	Overall (*N* = 1733)				*p*-value
	Physical activity					Physical activity					Physical activity				
	Moderate		Vigorous			Moderate			Vigorous		Moderate		Vigorous		
	OR^a^	95%-CI	OR^a^	95%-CI		OR^a^	95%-CI	OR^a^	95%-CI		OR^a^	95%-CI	OR^a^	95%-CI	
Gender					0.351					0.154					**0.050**
Men	1.00		1.00			1.00		1.00			1.00		1.00		
Women	0.89	0.61-1.32	0.71	0.44-1.14		0.91	0.62-1.35	**0.63**	0.38-1.01		0.91	0.69-1.19	0.66*	0.47-0.93	
Age					0.360					**<0.001**					**<0.001**
65 and above	1.00		1.00			1.00		1.00			1.00		1.00	2.07-8.13	
35-44	1.40	0.75-2.61	2.31	0.83-6.44		2.66***	1.53-4.64	6.12***	2.40-15.62		2.03***	1.36-3.05	4.06***	2.05-8.05	
45-54	1.31	0.70-2.48	2.47	0.88-6.94		2.89***	1.65-5.09	3.29*	1.24-8.70		2.01***	1.33-3.03	3.12***	1.59-6.28	
55-64	1.69	0.91-3.12	3.12*	1.13-8.60		2.26**	1.32-3.86	2.98*	1.15-7.75		1.99***	1.34-2.96	3.00***	1.49-6.103	
Education level					0.072					0.292					0.116
Tertiary	1.00		1.00			1.00		1.00			1.00		1.00		
Primary	1.68	0.82-3.42	1.06	0.43-2.64		0.42	0.11-1.56	0.54	0.12-2.43		1.26	0.70-2.27	1.05	0.51-2.15	
Secondary	2.06*	1.08-3.92	1.87	0.85-4.08		0.44	0.12-1.69	0.63	0.14-2.73		1.43	0.82-2.51	1.49	0.76-2.91	
Marital status					**0.041**					0.797					0.126
Divorced	1.00		1.00			1.00		1.00			1.00		1.00		
Single	**1.66**	0.99-2.78	2.10*	1.03-4.28		0.95	0.60-1.50	1.35	0.72-2.53		1.23	0.88-1.72	1.69*	1.06-2.69	
Married	1.24	0.74-2.07	1.09	0.53-2.26		1.02	0.68-1.53	1.28	0.72-2.30		1.10	0.81-1.51	1.20	0.77-1.88	
Occupation					**<0.001**					**<0.001**					**<0.001**
Less skilled	1.00		1.00			1.00		1.00			1.00		1.00		
Skilled	0.84	0.54-1.32	1.69*	1.00-2.85		1.23	0.73-2.06	2.70***	1.51-4.83		0.99	0.71-1.39	2.07***	1.41-3.05	
Location															**<0.001**
Rural											1.00		1.00		
Urban											1.67***	1.31-2.13	1.20	0.87-1.65	

*OR* odds ratios, *CI* confidence interval; *p < 0.05; **p < 0.01; ***p < 0.001; ^a^ Odds ratios adjusted for all variables in the table; bold *p* = significant borderline

Overall, women were 34% less likely (OR = 0.66, 95%-CI = 0.47-0.95) to engage in vigorous PA than men. The odds of engaging in vigorous PA decreased with increasing age, with the effects being significant across age strata overall and in urban and rural participants. Each age category was more likely to engage in moderate and vigorous PA than those in the category aged 65 and above, in both the overall cohort and in rural participants, but not in the urban ones (*p* < 0.001 for age*location interaction). Having a secondary education (relative to tertiary) was associated with an OR of 2.06 (95% CI = 1.08-3.92) for engaging in moderate PA among urban participants only.

Marital status was variably associated with PA in the overall cohort and across sites (*p* < 0.001 for the interaction marital status*location). For instance, single participants (relative to those divorced) were more likely to engage in vigorous PA in the overall cohort and in the urban site, Table 3.

In the overall cohort, skilled participants (relative to less skilled) were associated with higher odds of engaging in vigorous PA. The effect was similar in both rural and urban participants (*p* < 0.001 for occupation*location interaction).

## Discussion

This study provides insight into the socio-demographic correlates of PA levels in the urban and rural communities of South African adults. Over half the participants sampled engaged in moderate to high PA. However, a higher proportion of physical inactivity was observed in the rural participants compared to those in urban areas. Urban participants were more likely to meet recommended PA guidelines for public health than their rural counterparts. Similarly, the odds of participants achieving recommended PA guidelines (moderate PA) were 76% higher in an urban than in a rural setting. The results of this study can be compared with the findings of a PA survey from 22 African countries where prevalence of PA ranged from 72.5% (Swaziland) to 96.0% (Mozambique) [8]. Similarly, 67% of urban dwelling black South African women were classified as physically active [24]. Conversely, the odds of participants being physically inactive in United States was 43% higher in the extreme rural areas compared with urban ones [12]. A study in Cameroon, however, showed that rural dwellers were significantly more active than their urban counterparts based on objectively measured physical activity [25].

A high prevalence of physical inactivity in rural areas, especially in South Africa, may be because PA is largely of low intensity there. A study conducted in KwaZulu-Natal in South Africa reported high volumes of low intensity physical activity among rural children and adolescents [26]. It is also stated that the spread of technology used across different domains of society and the shift in the predominant type of employment and lifestyle behaviour, specifically from agriculture to industries and services, contributed to a reduction in physical activity [27]. The variations of results across studies could be due to different tools used to measure physical activity. In addition, there is difficulty to understand the intent of the IPAQ questions, in recalling the information requested, and in making the calculations required to perform physical activity [28]. Furthermore, different types of PA are undertaken between and within communities that are socially, economically, geographically and religiously different across aspects of life [29]. For this reason, objective assessment of physical activity would provide more insight in the levels and patterns of physical activity in South African population. Therefore, results of the current study are interpreted with caution.

The overall prevalence of moderate to vigorous physical activity did not differ significantly between genders. However, the adjusted odds ratio showed that women were 34% less likely to engage in the vigorous PA than men. A similar study found that women exhibited higher levels of inactivity than men and that inactivity was higher among older people [8]. Concurrently, a study conducted in Spain reported comparable results [30]. Conversely, studies conducted in Rwanda [31] and Nigeria [17], showed a higher prevalence of PA among women than in men.

Although it is difficult to interpret these conflicting results, South Africa could be described as a country in transition, and consequently there are currently high levels of infrastructure development, for which men are typically employed and often requires high levels of vigorous activity. Similarly, African cultural influence may make women more likely to be employed in domestic work such as cleaning and organizing households, which may mean they walk less, particularly in black townships areas [32]. Generally in African culture, women have hobbies that tend to keep them at home while men tend to engage in more physical recreational activities [32]. Based on these cultural differences, physical inactivity poses a major health risk to the South African women and with the projected increase in health risk to over 65 s, an increase in morbidity and mortality in these areas is expected. Hence, necessary interventions need to be implemented, among women through all age groups at societal level [33].

Our study showed that PA decreased with age, in accordance with previous studies [34, 35]. A similar study found that the volume of sedentary behaviour increased, whereas ambulatory activity [36] and recreational activity [37] decreased with age. Due to the fact that studies use different measures and definitions, direct comparisons are not possible. For example, vigorous activity have been reported to decrease with age, whereas moderate intensity activity increase from ages 13 to 27 years [38]. Furthermore, the relationship can be affected by the effect of various confounding factors such as genetic, cultural, socio-economic, nutritional factors and inactivity [39]. These act through reduction of the functional abilities, strength, and ambulation associated with increased age-related diseases. As a result, the relationship between physical activity and healthy ageing among adults still remains complex, and physical activity levels must be taken into account in ageing studies [39].

Level of education has also been associated with PA [40]. Similarly, results in this study showed that in urban areas, participants who had reached secondary level education were twice more likely to engage in moderate PA than those with tertiary education. In rural areas, participants with primary education tended to be the least likely group to engage in moderate PA. These results are comparable to another study which found the level of education was associated with the likelihood of walking [41]. Thus, people with less education may be more likely to walk or cycle than the highly educated, possibly because the latter may own a car, with the associated reduction in physical inactivity. It is also likely that less well educated people may be employed in jobs that are more physically demanding, while they also have insufficient money and time to engage in leisure-time PA. Contrarily, people at a higher educational level may have more sedentary jobs, but may engage more in leisure-time PA that those less well educated because they are more aware of it and its associated health benefits [42].

Cross-sectional studies report mixed results concerning the relationship between marital status and PA, although it is often an inverse relationship, where married individuals are less physically active than those who are unmarried [43]. This study showed that, both in the urban site and overall, a higher proportion of single participants engaged in moderate-to-vigorous PA than those who were divorced or separated, and comparable results to these were reported by a study in Lebanon [44]. There are however also reports that show married people as being more active than the single people [45], and a study based in Nigeria found a positive association between being married and reaching sufficient PA levels [18]. However, in this study, being married was not a determinant factor for PA.

Contrasting results in studies of the relationship between marital status and PA could be a consequence of contrasting variables. For instance, cultural expectations of married African adults, especially men, may differ and men may be expected to be the primary earners [18] but this may not be true in all countries, such as South Africa.

Likewise, this study noted that participants with skilled jobs were more likely to engage in vigorous PA than less skilled (or homemakers). Similarly, a study in Mexico showed that a higher percentage of adults working in agriculture and fishing were in a higher activity level category than those in lower-intensity occupational activities, the latter also having a greater proportion of participants in low and moderate activity levels [46]. A study in Australia, however, showed those participants in the lower strata of occupations to be less likely to report participation in vigorous PA sufficient to achieve cardiorespiratory fitness [47]. Most of sub-Saharan African countries, particularly those undergoing rapid developments, are in the midst of demographic and epidemiologic transitions. These developmental processes bring about changes in the social capital of societies, change working patterns and lifestyles contributing to reduction in physical activity levels [48].

### Limitations and strengths

Our study has some limitations. Its cross-sectional design did not allow for the investigation of causal relationship among characteristics. This study was restricted to adults only, in two provinces of South Africa, and its findings may not be applicable countrywide. Finally, another limiting factor of this study was that PA was assessed with a version of IPAQ, a self-report measure associated with overestimation of PA levels [45]. Nevertheless, this study was based on a large cohort of urban and rural South Africans, primarily of African descent which was assessed using a standardized method for surveying risk factors for chronic diseases. The study adds to previous reports by providing determinants and prevalence of PA levels in an urban and rural setting in South Africa. However, future studies aiming at monitoring of the exposure to PA should consider conducting objective assessment of PA in order to validate PA in urban and rural communities.

## Conclusion

Culturally or community tailored intervention to promote physical activity should target individuals at an early age, those with primary, tertiary education, married and divorced and rural residents in South Africa. The current study indicates that if no effective public health approach or social economic plans are implemented, further decrease in physical activity will lead to high risk of developing major chronic diseases among South Africans. Studies using objective assessment of physical activity are needed to confirm these findings.

## Abbreviations

BMI: Body mass index
IPAQ: International Physical Activity Questionnaire
METs: Metabolic rate
NCDs: Non-communicable diseases
PA: Physical activity
PHRI: Population Health Research Institute
PURE: Prospective Urban and Rural epidemiology study
USA: United Sates of America
UWC: University of the Western Cape
